# Multi-Level Seg-Unet Model with Global and Patch-Based X-ray Images for Knee Bone Tumor Detection

**DOI:** 10.3390/diagnostics11040691

**Published:** 2021-04-13

**Authors:** Nhu-Tai Do, Sung-Taek Jung, Hyung-Jeong Yang, Soo-Hyung Kim

**Affiliations:** 1 Department of Artificial Intelligence Convergence, Chonnam National University, 77 Yongbong-ro, Gwangju 500-757, Korea; donhutai@gmail.com (N.-T.D.); hjyang@jnu.ac.kr (H.-J.Y.); 2 Department of Orthopedic, Chonnam National University Medical School, 160 Baekseo-ro, Gwangju 61469, Korea; stjung@jnu.ac.kr

**Keywords:** knee bone, tumor, cancer, u-net, segnet, deep learning, segmentation, classification, detection

## Abstract

Tumor classification and segmentation problems have attracted interest in recent years. In contrast to the abundance of studies examining brain, lung, and liver cancers, there has been a lack of studies using deep learning to classify and segment knee bone tumors. In this study, our objective is to assist physicians in radiographic interpretation to detect and classify knee bone regions in terms of whether they are normal, begin-tumor, or malignant-tumor regions. We proposed the Seg-Unet model with global and patched-based approaches to deal with challenges involving the small size, appearance variety, and uncommon nature of bone lesions. Our model contains classification, tumor segmentation, and high-risk region segmentation branches to learn mutual benefits among the global context on the whole image and the local texture at every pixel. The patch-based model improves our performance in malignant-tumor detection. We built the knee bone tumor dataset supported by the physicians of Chonnam National University Hospital (CNUH). Experiments on the dataset demonstrate that our method achieves better performance than other methods with an accuracy of 99.05% for the classification and an average Mean IoU of 84.84% for segmentation. Our results showed a significant contribution to help the physicians in knee bone tumor detection.

## 1. Introduction

Knee tumors are tumors that appear around the bone regions of the human knee; they often fall into three types: benign, malignant, and pseudo-tumors. These tumors inflict substantial physical and emotional pain on the affected patients. Early clinical investigations used conventional radiographs to detect knee injuries, as they were an effective tool that was also cheap. Despite the excellent assistance provided by radiographic images, the manual processes involved in collecting one’s medical history, imaging examination, and image analysis are often time-consuming, which delays the development of an appropriate treatment plan, and which can lead to disability or death for the patients. However, we believe that computer-aided diagnostics can be a valuable tool that helps clinicians make correct and timely decisions. As a result, patients will eventually have more reliable diagnoses, leading to the earlier initiation of appropriate treatment, and, consequently, a prolonged life.

Recently, the automation of conventional radiographs analysis using convolutional neural networks (CNNs) [1,2,3] has emerged as a breakthrough research field in general visual recognition, which is particularly useful for medical images. Although X-ray images suffer from noises, the segmentation approach is very effective in reducing noise. Thus, these deep learning models can already achieve high performance in medical image segmentation. This clearly shows the possibility of applying them in clinical practice. The disadvantage is that these models must be trained on huge datasets to be able to make predictions. However, the number of publicly available medical datasets is limited, especially on datasets of the knee bone. Therefore, we must first prepare a quality dataset of knee bone tumors before starting to build the system.

**Figure 1:**
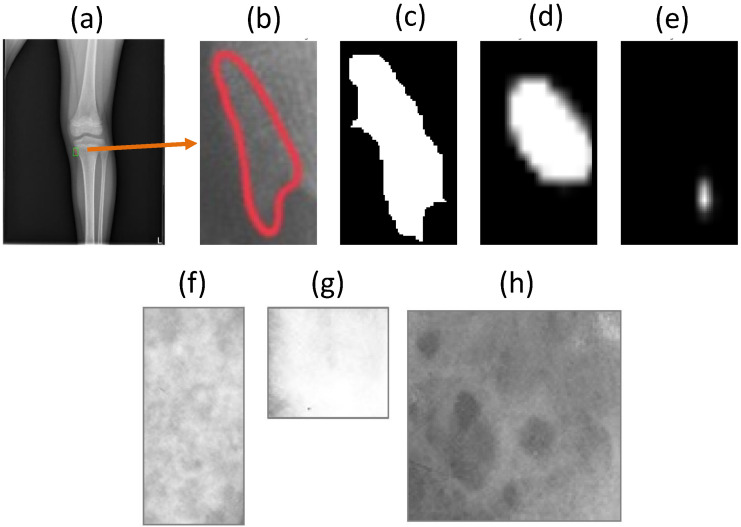
(Row 1) The robust multi-level features used to help detect the very small tumor region. From left to right, (**a**) original image containing the very small tumor region, (**b**) zoomed-in view of the tumor region, (**c**) the ground-truth, (**d**) the result with multi-level distance features, and (**e**) the result without those features. (Row 2) Meaningful bone tumor matrices of knee bone tumor classification shown in (**f**–**h**). It is proven to be a highly predictive feature of bone tumor classification in [5]. This explains why global and patch-based approaches should be applied to distinguish between benign-tumor and malignant-tumor regions.

Our dataset is collected from various sources, and it is supported directly by many physicians. However, we encountered many difficulties in the data normalization of knee X-ray images because of data heterogeneity; for example, tumors can appear anywhere in the knee area, so the posture also changes. The process of setting and adjusting parameters for radiography can also vary significantly from one dataset to another. For example, a patient examined in one hospital may have different knee X-ray images in another hospital. In addition, the raw knee X-ray images are often high-resolution and of extremely massive sizes, thereby requiring substantial amounts of memory for the training process. It is therefore necessary to scale down the radiology image in pre-processing. Consequently, under the resize-image condition, some small tumors almost disappear, while others become too small, as shown in Figure 1. However, Vartevan et al. [4] described a way to recognize tumors by margins, periosteal reaction, bone destruction, and the existence of a soft-tissue mass. These local textures can help detect tumors of any size, and they should be added to the learning process of the machine. In 2018, Reicher et al. [5] used a tumor matrix to only discriminate the local features of tumors in the radiology images, as shown in Figure 1f–h. More details in related works are mentioned in Appendix A.

Unlike other research using global information to detect, classify, and segment tumors [6], our model combines both global and patched-based approaches using muli-level distance features. In the global-based approach, our Seg-Unet model aims to not only learn the whole geometric context of the knee bone, but also exploit high-risk regions for tumor occurrence. For example, tumor regions commonly have a small size, unclear border, and uncommon appearance. Similar to the diagnosis process, our model assesses the whole X-ray images and learns the texture regions around the high-risk regions near the tumor regions. Through the learning processes, our model in practice will have the ability to efficiently focus on the tumor regions based on the global geometric characteristics of the knee bone as well as the textures of the high-risk regions, where they are larger than the tumor regions.

In the patch-based approach, our model is transferred the weight from the global model, and image patches are input into the model for further fine-tuning. In contrast to the whole radiology image used in the global model, the patch model focuses on image patches consisting of small image chunks of the high-resolution original image. These help our patch model deal with malignant tumor regions which have the most uncommon appearance and the smallest size. From transfer learning by the global model, the patch model integrates the global information of the whole image with the local information of small regions to boost the performance of malignant tumor detection.

Our contribution in this study is to propose a multi-level Seg-Unet model using a combined global and patch-based approach to deal with small tumor regions and achieve improvements in malignant tumor detection. Our model has an encoder–decoder architecture that leverages the mutual benefits of classification and segmentation branches to learn the global geometric context and local texture features at every pixel. Moreover, the multi-level distance features help improve our model’s performance in high-risk places around tumor regions. In addition, the patch-based aspect uses the weight of the global-based model to make suitable fine-tuning to detect malignant tumor regions from small image chunks of the original high-resolution image. Finally, we build and conduct experiments on a knee bone tumor dataset with the annotations of physicians at Chonnam University Hospital (CNUH). We hope to contribute to knee bone tumor research and the use of deep learning to address various issues.

The rest of our paper is organized as follows: In Section 2, we present the materials and our proposed method for knee bone segmentation. Next, we provide our results in Section 3 and the discussion in Section 4. Finally, in Section 5, we conclude our research. Related works, implementation details, environment setup, and evaluation metrics are mentioned in the Appendix A, Appendix B and Appendix C.

## 2. Materials and Methods

### 2.1. CNUH Dataset and Challenges

In this study, we evaluated our method on the knee bone tumor dataset Chonnam National University Hospital (CNUH) [7] presented in Table 1. This dataset includes 1195 tumor images and 381 normal images. Institutional review board approval was obtained. The condition for informed consent was waived as this study was a retrospective review of radiologic images without demographic data of patients.

**Figure 2:**
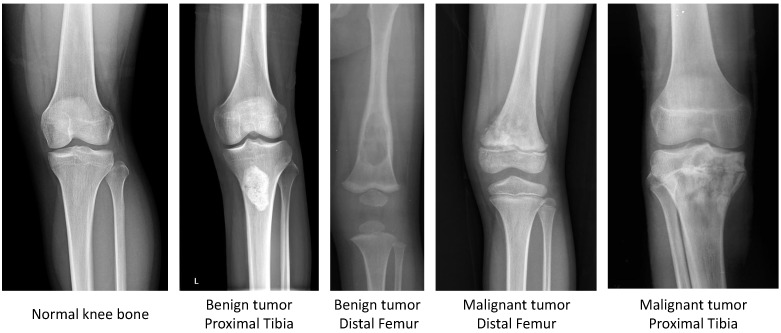
Sample images in the CNUH Dataset.

Our dataset focuses on benign and malignant tumors in two regions of knee bone, i.e., Distal femur and Proximal tibia, as shown in Figure 2.

**Figure 3:**
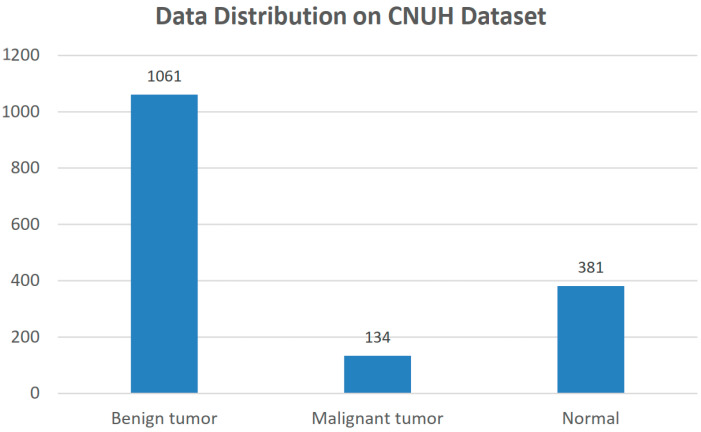
Data Distribution in the CNUH Dataset.

**Figure 4:**
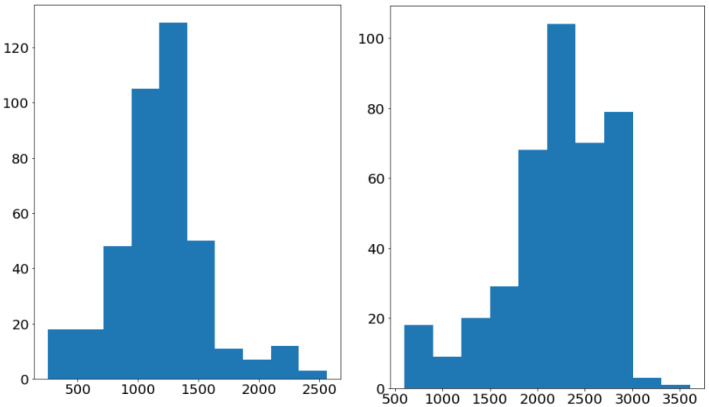
The weight (**left**) and height (**right**) distribution of the images in the CNUH Dataset.

Figure 3 illustrates the data distribution among three labels in our problem. There is an imbalance in our dataset where the number of benign tumors (1061 images) is larger than the number of malignant tumors (134 images). Almost all of the images are large, with a maximum size of 3480×4240 and a minimum size of 330×597, as shown in Figure 4. Otherwise, the tumor regions have a wide variety of sizes, ranging from the approximate small size of 100 to the approximate large size of 1500.

**Figure 5:**
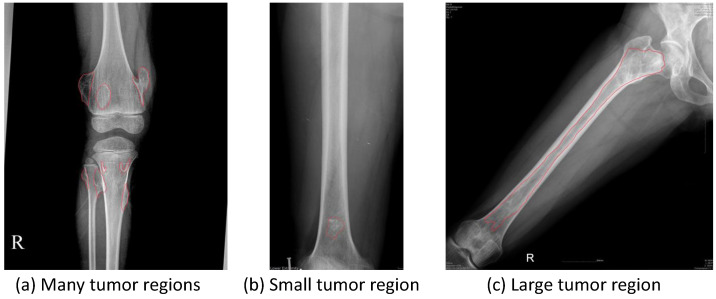
Difficult tumor images in CNUH dataset. The red lines are the ground-truths drawn by the CNUH physicians. These sample images show common challenges in the CNUH dataset such as (**a**) diversity of tumor occurrence, (**b**) too small size of tumor region, and (**c**) too large size of tumor region and pose variety.

Figure 5 illustrates the challenges faced in knee bone tumor detection. The imbalance in the number of tumor-malignant images leads to difficulties in tumor detection based on the limited data. In addition, the imbalance between tumor regions and background regions also leads to a reduced performance of tumor detection in practice. Moreover, some difficult cases often arise, such as a high diversity of sizes; the number of tumor regions, which can vary from a minimum of 1 to a maximum of 8; and the range of changes in tumor sizes, from very small regions leading to important distortions when zooming out to very large regions covering almost the entire image, which prevent the accurate detection of the full tumor region.

Therefore, the goal of this study is to propose a robust method by which to detect normal and tumor regions as well as classify knee bone images among three labels (normal, benign, and malignant) to tackle the challenges described above. It is expected to be a useful recommendation application to help physicians diagnose knee bone tumors early.

### 2.2. Proposed Method

#### 2.2.1. Overview

In this study, our input is a scaled-down or patch image $X\in R^{H\times W}$ of the human knee region in an X-ray image with width *W* and height *H*. Our first task is to determine the one-hot tumor probability $Y_{clas}\in R^{C}$ to determine the specific tumor label $y_{c}\in \left\{0,1,2\right\}$ corresponding to the normal, benign, or malignant label. Let $p≜\left(x,y\right)$ be the pixel location of the given X-ray image X. The second task is to segment the tumor regions in the X-ray image *X*, then output the tumor segmentation mask $Y_{seg}\in R^{W\times H\times 2}$ where the pixel-tumor probabilities $Y\left(p\right)$ at every pixel *p* in *X* determine whether it belongs to the normal or tumor label. In this study, we suggest a third task of determining the multi-level distance map $Y_{dist}\in R^{W\times H\times 5}$ to exploit the high-risk regions around the tumor regions. Every pixel-level distance feature $Y_{dist}\left(p\right)$ helps a physician by providing five levels of alert, consisting of normal, tumor, and high-risk tumor levels from 1 to 3, depending on the distance to tumor. The third task creates an attention map to enhance our performance in difficult cases under in-the-wild conditions such as small tumor regions and malignant tumors. Therefore, our problem becomes finding the knee bone detection model M including three mapping functions $F_{clas}$, $F_{seg}$ and $F_{dist}$ to predict the tumor probability ${\hat{Y}}_{clas}$ in the whole image, as well as the tumor segmentation mask ${\hat{Y}}_{seg}$ and the multi-level distance map ${\hat{Y}}_{dist}$ to identify the tumor regions and high-risk tumor regions as follows:

(1) $$\begin{array}{c} \begin{array}{cc} M & \;=\;\left\{F_{clas},F_{seg},F_{dist}\right\} \end{array} \end{array}$$

where:

(2) $$\begin{array}{c} \begin{array}{cc} {\hat{Y}}_{clas} & =F_{clas}\left(X\right)\\ {\hat{Y}}_{seg} & =F_{seg}\left(X\right)\\ {\hat{Y}}_{dist} & =F_{dist}\left(X\right) \end{array} \end{array}$$

**Figure 6:**
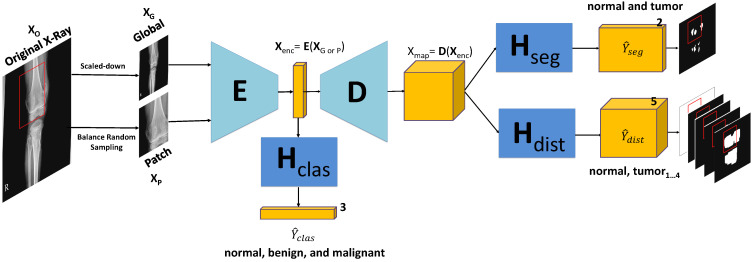
Knee Bone Tumor Detection Model. It uses the geometric-context classification $H_{clas}$ to enhance the encoding feature $X_{enc}$ from encoding *E* to return the geometric-context probability vector $Y_{clas}$. The 2D decoding feature map is enhanced by multi-task learning at the pixel level between the pixel-tumor segmentation $H_{seg}$ and the high-risk pixel-tumor segmentation $H_{dis}$ for outputting tumor segmentation mask ${\hat{Y}}_{seg}$ and multi-level distance features ${\hat{Y}}_{dist}$. ${\hat{Y}}_{dist}$ has the role of high-risk attention around tumor regions. The model’s input is from the scaled-down image $X_{G}$ of the original X-ray image *X* for the global model and the patch image XP generated from balance random sampling for the patch model.

As shown in Figure 6, our model M contains the encoding block E, the decoding block D and three branch blocks $H_{clas}$, $H_{seg}$ and $H_{dist}$ for multi-task learning. The goal of multi-task learning is to provide mutual information to enhance the performance of our proposed models by encoding feature $X_{enc}$ and decoding feature map $X_{map}$.

Due to the complexity of knee bone X-ray images under challenging conditions such as the various potential poses, size diversity, and uncommon appearance, we need the classification branch to determine at the global-context level whether an image belongs to normal or tumor (including benign and malignant). It is placed at the middle of the model to provide information to the encoding feature $X_{enc}$ to improve the encoding block *E* as follows:

(3) $$\begin{array}{c} \begin{array}{cc} {\hat{Y}}_{clas} & =H_{clas}\left(E\left(X\right)\right)=F_{clas}\left(X\right) \end{array} \end{array}$$

where $X_{enc}=E\left(X\right)$ compacts the original image X into the features to be calculated for classification, segmentation, and high-risk segmentation. This then serves as the input of decoding block *D* to calculate the results for the pixel-level tasks in the two remaining branches as follows:

(4) $$\begin{array}{c} \begin{array}{cc} {\hat{Y}}_{seg} & =H_{seg}\left(D\left(X_{\mathrm{enc}}\right)\right)=F_{seg}\left(X\right)\\ {\hat{Y}}_{dist} & =H_{dist}\left(D\left(X_{\mathrm{enc}}\right)\right)=F_{dist}\left(X\right) \end{array} \end{array}$$

where $X_{map}=D\left(X_{enc}\right)$ is the decoding feature map for the pixel-level feature representation affected by the tumor segmentation and high-risk tumor segmentation branches. This means that the tumor segmentation branch can efficiently learn the distance information of the high-risk tumor segmentation branch and the global-context feature of the classification branch under challenging conditions. Otherwise, the classification branch is also enhanced from the mutual information. The distance feature calculation is described in further detail in the section below.

#### 2.2.2. Global and Patch-Based Models

Our proposed system involves two approaches derived from the above model M consisting of a global-based model $M_{G}$ and a patch-based model $M_{P}$. The global model $M_{G}$ receives the high-resolution image $X_{O}\in R^{W_{O}\times H_{O}}$ with $W_{O},H_{O}\leq 4000$ and scales it down into a small image $X_{G}\in R^{W\times H}$ with a suitable size W,H. It then uses multi-task learning to learn the mutual information from three tasks: classification, tumor segmentation, and high-risk tumor segmentation. From there, it can not only detect small tumor regions but also alert physicians to the high-risk regions around tumors.

Meanwhile, the patch-based model $M_{P}$ makes predictions from a small image chunk $X_{P}\in R^{W\times H}$, where $X_{P}$ is cropped from $X_{O}$. The image chunks fed to $M_{P}$ will be generated by balance random sampling which obtains small regions in the high-resolution original image that satisfy the balance constraint among normal and tumor region occurrences. By transferring the weight from the global model $X_{G}$, $X_{P}$ takes advantage of the knowledge of the geometric and texture features at the whole image level to apply it in small images chunks for detecting difficult cases in tumor detection, particularly malignant tumor detection.

However, global and patch-based models both have specific advantages and disadvantages. For examples, the global-based model $M_{G}$ faces difficulties when its inputs are very high-resolution X-ray images. In this study, our image size is almost the width of 3000 and the height of 4000, while the width and height of the tumor regions are commonly small, with a value of about from 100 to 1000 for each. The global-based model with multi-level distance features is almost as good at segmentation of normal and tumor regions, but it faces difficulties when distinguishing between benign and malignant regions. This means that it is good for learning geometric features and large texture regions. By contrast, our patch-based model $M_{P}$ tackles difficult problems that arise in learning texture features from small image chunks. It also uses detailed multi-level distance features to identify uncommon appearances and the smallest regions in which malignant tumors often appear. However, the above advantages lead to difficulties for the patch-based model in classifying among normal and tumor regions due to its sensitivity in tumor detection; it often fails in false-positive cases.

Therefore, we proposed a method using a combination of global and patch-based models for the segmentation and classification problem. For the classification problem, we use the results of normal and begin prediction from the global-based model, as well as the malignant prediction from the patch-based model. For the segmentation problem, we use the weighted average method to integrate the results of the global and patch-based models.

More details are mentioned in Appendix B.

#### 2.2.3. Model Architecture Details

**Figure 7:**
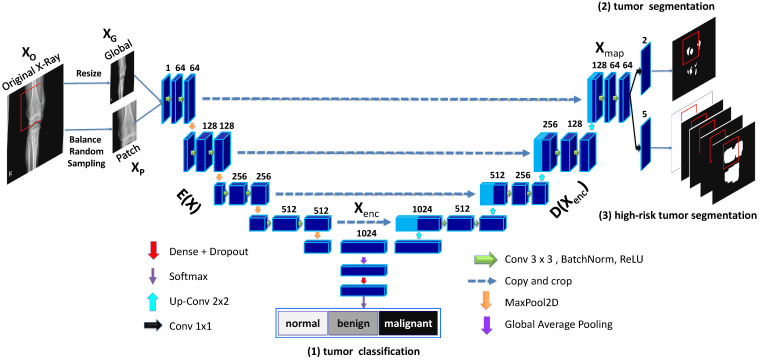
Multi-level Seg-Unet model with global and patch-based approaches.

Our network architecture is illustrated in further detail in Figure 7 with the global and patch-based approaches. The input of our model is a down-scale image $X_{G}$ or an image patch $X_{P}$ from an original high-resolution image $X_{O}$. There are three outputs in our model, including the classification result ${\hat{Y}}_{clas}$, the tumor segmentation result ${\hat{Y}}_{seg}$, and multi-level high-risk tumor result ${\hat{Y}}_{dist}$. ${\hat{Y}}_{clas}$ is the one-hot probability vector used to determine whether the input belongs to one of the normal, benign, or malignant labels. ${\hat{Y}}_{seg}$ with size W×H×2 is used to classify whether each pixel of the input belongs to normal or tumor. Finally, ${\hat{Y}}_{dist}$ with size W×H×5 determines the attention level among normal, tumor, or high-risk from levels 1 to 3 based on the distance to tumor.

We choose the Seg-Unet architecture [7] based on U-Net [8] with contracting and expanding paths, as well as Seg-Net [9] with the pooling-indices layer. The contracting path at the left side of the model has the encoding block $E\left(X_{enc}\right)$ with the goal of encoding features $X_{enc}$ from the input X. At the middle location, the classification branch uses the global average pooling to extract the encoding feature followed by dense and soft-max layers to classify the input into normal, benign, or malignant labels based on the classification probability vector ${\hat{Y}}_{class}$. Next, the right side of the model is the expanding path corresponding to the decoding block $D\left(X_{enc}\right)$, which maps the encoding feature into a decoding feature map $X_{map}$ at the pixel-level. From there, two remaining branches can be mapped into the tumor segmentation map ${\hat{Y}}_{seg}$ and the high-risk tumor segmentation map ${\hat{Y}}_{dist}$.

### 2.3. Experiments Setup

**Figure 8:**
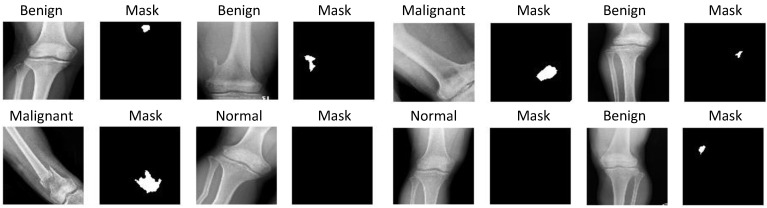
Data augmentation in the global-based model with transform operators such as resizing, rotating, center cropping, and flipping randomly.

**Figure 9:**
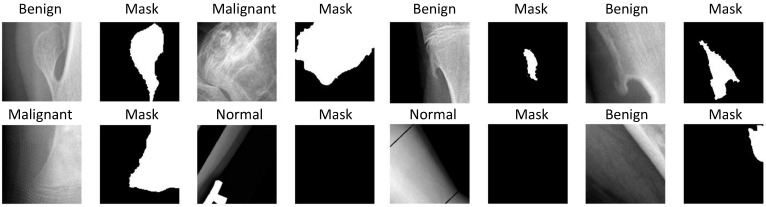
Data augmentation in the patch-based model with transform operators such as resizing, rotating, center cropping, and flipping randomly.

Training Process. We separated the CNUH dataset into two subsets comprising training data and validation data with the ratio value of 80/20. For the global-based model, we resized the image to 416×416 and randomly applied rotation, flipping, or cropping for augmentation, as shown in Figure 8. For the patch-based model, we used balance random sampling to obtain sub-regions of the original image with the size 416×416. The sampling process had constraints such as balancing between the tumor and normal regions with a main focus on bone regions. We assigned the normal region as the area where the tumor mask was too small below the specified number of pixels. We then applied data augmentation on the sub-regions in the same way as the data augmentation in the global-based model shown in Figure 9.

First, we trained the global-based model using Adam optimization with a learning rate of 0.001 [10] while reducing on the plateau and stopping early after 20 epochs. Next, we used the pre-trained weight of the global-based model for the weight initialization of the patch-based model. We trained the patch-based model by SGD optimization [11] with a learning of 0.0004 while reducing on the plateau and stopping early after 20 epochs.

Ablation Study. For the specific evaluation of the effects of different parts in our proposed models, we adjusted our proposed model as follows: with/without classification branch, tumor segmentation branch, and high-risk tumor segmentation branch (also called multi-level distance) under global, patch, and combination approaches. There are five deviation models in total in the ablation-study experiment, as listed in Table 2. More details in environment setup and evaluation metrics are mentioned at Appendix C.

## 3. Results

### 3.1. Experiments on Tumor Segmentation

We conducted experiments on five models (numbered from 1 to 5) by adjusting for the use and non-use of segmentation and multi-level distance branches in the global and patch-based approaches. Table 3 presented our quantitative results with the MeanIoU metric as follows:

For the tumor classification branch, our model learned the global-context feature from the whole X-ray image to improve the encoding feature $X_{enc}$. This helped Model 2 increase the tumor segmentation result from the MeanIoU of 69.50% obtained in Model 1 to the MeanIoU of 77.28% obtained in Model 2; this is the significant increase of 7.78%.

**Figure 10:**
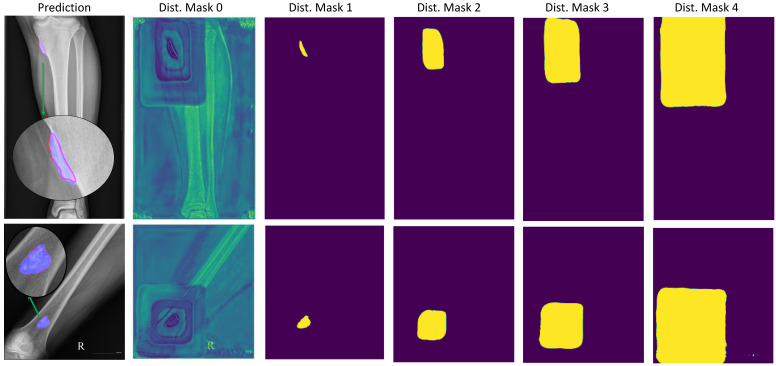
Tumor segmentation result with segmentation branch and high-risk tumor segmentation result with multi-level distance branch. The prediction column shows the original image with a red line for the ground-truth and a blue mask for the tumor mask prediction. The multi-level distance mask has five level masks, with distance mask 0 for the background region, 1 for the tumor region, and 2 through 4 for the three distance levels corresponding to tumor regions.

For the multi-level distance branch, the multi-level distance feature map helped our model recognize small tumors based on neighbouring regions called high-risk regions, with three levels based on a image distance percentage of 0.25, 0.5, or 0.75. This provided a slight increase of 1.55% in Model 4 from Model 2 by enhancing the decoding feature map $X_{dec}$ based on the multi-level distance map shown in Figure 10.

There are difficult cases in which the tumors are very small compared to the background region. Our model attempted to detect small tumors by learning mutual information from the neighboring feature maps around tumors. Figure 10 illustrates the learning result from the neighboring feature maps in column 2. The figure shows the background probabilities in the distance feature map where there were four rectangles from nearest to furthest to show four levels of distance to the tumor position.

Finally, one of the challenges in knee bone tumor detection is the size of the high-resolution image in contrast to the very small size of the tumors. The image input is often resized to be suitable for our global-based model due to its limited memory. This leads to a loss of important image texture for recognizing tumors, especially small tumors. This problem is fixed by the patch-based model learning detailed image texture from image patches. By contrast, the patch-based model is improved by the global-based model from the geometric characteristics on the whole image. Therefore, the performance of Model 5, which is a fusion between Models 3 and 4, leads to a significant increase of 6.95% with a MeanIoU of 84.84%.

We conducted a detailed analysis of the successful and failed cases in the fusion method of both the global and patch-based models. In the figures below, the red lines represent the ground-truths according to physicians while the blue regions show the detection results.

**Figure 11:**
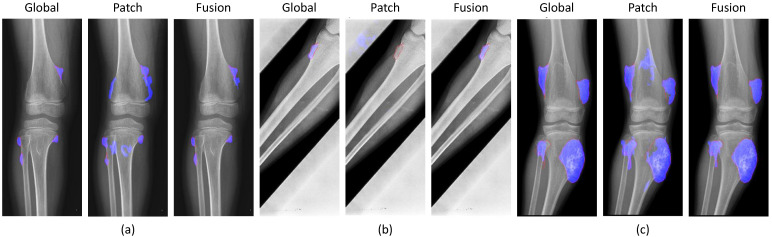
Successful cases of the global-based model enhancing the fusion results with (**a**) small tumors, (**b**) variant poses, and (**c**) large tumors. (red line: ground-truth, blue region: tumor detection).

Figure 11 shows the results of the successful cases using the fusion results from the global-based model. The patch-based model failed with (a) noise in small tumors, (b) non-tumor detection in variant pose, and (c) noise in larger tumors. However, the global-based model detected these successfully.

**Figure 12:**
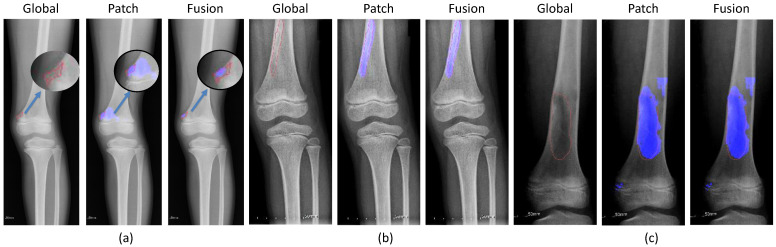
Successful cases of the patch-based model enhancing the fusion results with (**a**) small tumors, (**b**) long tumors, and (**c**) large tumors. (red line: ground-truth, blue region: tumor detection, blue arrow: zoom in the small ground-truth regions.)

By contrast, Figure 12 illustrates the successful prediction of the patch-based model with (a) small, (b) long, and (c) large tumors. The fusion of both models achieved good performance by integrating the results of the patch-based model.

**Figure 13:**
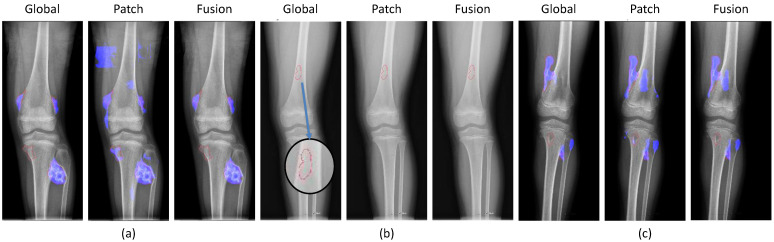
Failed cases of the fusion between global and patch-based models with (**a**) not enough tumor regions, (**b**) no tumors, and (**c**) not enough tumor and noise. (red line: ground-truth, blue region: tumor detection, blue arrow: zoom in the small ground-truth regions).

Finally, Figure 13 shows the failed cases of the fusion method. Here, the global and patch-based models met problems in tumor detection stemming from insufficient tumor detection, noise, and non-tumor detection.

### 3.2. Experiments on Tumor Classification

We also performed a classification evaluation on the CNUH dataset using four models in the ablation study, as presented in Table 4.

The fusion method of the global and patch-based models provided the best result with an Accuracy of 99.05% and a MeanAccuracy of 96.30% compared to Model 2 (only using classification and segmentation branches) with values of 95.27% (Accuracy) and 82.27% (MeanAccuracy), as well as Model 4 (addition of multi-level distance feature) with values of 94.32% (Accuracy) and 96.30% (MeanAccuracy).

**Figure 14:**
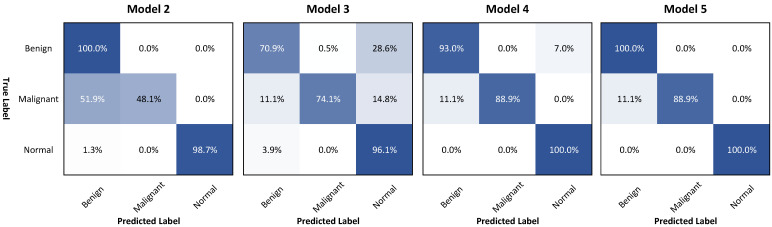
Confusion matrices of four models used in the classification experiment.

To explain this, Figure 14 points to the important improvement in the accuracy of malignant classification when using the multi-level distance feature map from 48.1% in Model 2 to 88.9% in Model 4 by the confusion matrix analysis. Although the overall accuracy of Model 4 (94.3%) was lower than the accuracy of Model 2 (95.27%), the mean accuracy of Model 4 (93.96%) was increased faster than the mean accuracy of Model 2 (82.27%). By integrating the global and patch-based approaches, we slightly enhanced the accuracy of the benign performance in the global-based model 4 from 93.0% to 100% in Model 5 (the fusion of both models).

## 4. Discussion

**Figure 15:**
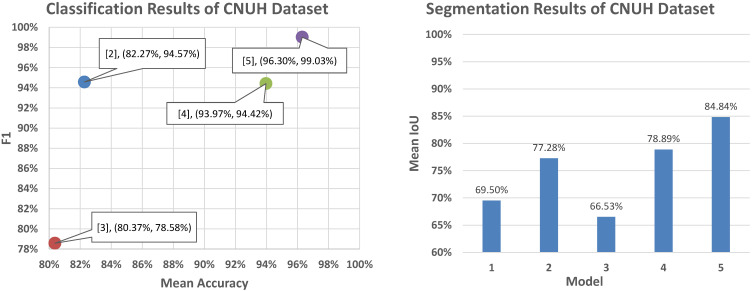
Classification and segmentation performance in the CNUH validation set.

Figure 15 presents our classification and segmentation experiments in the ablation study. This research illustrated the important role of multi-task learning of learning mutual information between the classification branch for the global context and the pixel-level features with segmentation and multi-level distance features. For segmentation performance, Model 4 with three branches achieved a good performance of 78.89% compared to those of Model 1 (only using segmentation) of 69.50% and of Model 2 (using classification and segmentation branches) of 77.28%. In addition, Model 4 provided good performance with a MeanAccuracy of 93.97% compared to the MeanAccuracy of 82.27% of Model 2. This represented a significant improvement in malignant accuracy with an accuracy of 88.9%, as shown in Figure 14.

In this study, the multi-level distance feature map served as an attention map to help our model detect small tumors. They provided the probability of background region, tumor region, and neighbouring regions around tumors. From there, the tumors can be recognized based on the pixel-level features of the neighbouring regions in difficult cases with very small tumors in high-resolution images. The background region feature map shown in Figure 10 illustrates the efficiency of learning the multi-level distance feature. It shows four rectangles corresponding to the four distance levels from distances 0 (at tumor), 0.25, 0.5 and 0.75 according to the ratio of image size.

To deal with the high-resolution image, the patch-based model received image patches of the original images to learn the detail texture. We used the pre-trained weight of the global-based model to transfer learning to the patch-based model to overcome the convergence problem and to transfer the global features of the whole X-ray image. Due to a lack of global information from the whole X-ray image, the patch-based model met problems such as sensitivity to benign and malignant tumors leading to false negatives in prediction processing, as shown in Figure 11. The accuracy values of the classification and segmentation in the patch-based model were 77.29% and 66.53%, respectively. However, it also improved the global-based models in difficult cases, as shown in Figure 12. From there, the fusion of both models achieved the best overall performance results in the classification and segmentation evaluations, with respective values of 99.05% and 84.84%.

Comparison with related works. Table 5 presents a comparison of the performance results of our proposed method with those of related stuides using the CNUH validation set.

For classification comparison, Huynh et al. [14] proposed a regenerative semi-supervised bidirectional W-network (RSS-BW) for classification into normal, benign tumor, and malignant tumor from the X-ray images. They used the encoder–decoder model to extract bone regions. From there, that model classified three types of tumor state based on the input image and bone regions. They achieved a classification performance of 86.93% with backbone VGG16. For comparisons on segmentation, we compared our model to related works [7,15,16]. We also included conventional models in classification and segmentation to train and evaluate; these were MobileNet V2 [12] and VGG16 [13] in classification evaluation and U-Net [8] and Seg-Net [9] in segmentation evaluation. Our proposed method achieved the best results in classification and segmentation with an Accuracy value of 99.05% and a MeanIoU value of 84.84%

Our work demonstrates that the Seg-Unet model with multi-level features can provide meaningful results for classifying and segmenting knee bone tumors in X-ray images. It is able to compare to the accuracy of 80% of the experienced experts in bone tumor detection [17]. We try to integrate it as a module in the medical imaging software such as Slicer3D, MITK Workbench to improve the diagnostic accuracy that decision support will benefit those with less experience. It is useful in clinical diagnosis using imaging modeling when the timely and accurate diagnosis is challenging dealing with non-specific symptoms that mimic common musculoskeletal injuries, late patient presentation, and low suspicion by physicians [18]. Moreover, it is helpful in the analysis of a potential bone tumor against difficult conditions such as the small size, appearance variety, variant pose, and high resolution. The current research is only for bone tumor detection around the knee region, but our system could be extended in the future for the bone tumors around the lung, arm, or another typical area for X-ray inspection.

## 5. Conclusions

In this paper, we proposed a Multi-Level Seg-Unet model with global and patch-based approaches for the detection of knee bone tumors from X-ray images. Our goal is to assist physicians in knee bone detection from radiology images by segmenting tumor regions and classifying them into three labels: normal, benign, and malignant. Our problem deals with challenges that arise in the knee bone tumor diagnosis process in the CNUH dataset such as small size, high-resolution, uncommon appearance, and variant pose for knee bone tumor detection on X-ray images.

Our proposed model employed multi-task learning with classification, tumor segmentation, and high-risk tumor segmentation using a multi-level distance feature map. Three branches help our model learn mutual information to enhance the global-context encoding feature vector and the pixel-level decoding feature map. The segmentation result of the model with three branches shows important increases of 1.61% and 9.39% over the models using two branches (segmentation and classification) and the segmentation branch alone, respectively. The classification result in MeanAccuracy was also increased to 93.97% for the model using three branches compared to the model using two branches, with 82.27%.

Moreover, the multi-level distance feature map at the high-risk tumor segmentation branch provides an important contribution to detecting tumors with a small size and uncommon appearance, and suggests a distance feature map for determining neighbouring regions around tumors. The malignant accuracy in the model using the multi-level distance map was 88.9%, compared to the value of 74.1% obtained with the model using only the classification and segmentation branches.

Finally, the patch-based model was transferred the weight from the global-based model to further fine-tune image patches to focus on texture details having information loss based on the small tumor size against the high-resolution image. From there, the fusion of global and patch-based models helped improve the classification and segmentation results. Our proposed method with the fusion of both models achieved the best performance, with a MeanIoU value of 84.84% in segmentation and a MeanAccuracy value of 99.05% in classification.

In the future, our proposed method needs to improve the performance of the global and patch-based models in an end-to-end fashion in training to better exploit the global-context features in the whole X-ray image as well as the texture details in image patches. We also need to exploit bone shape relating to tumor regions with a graph convolution neutral network as well as bone location with object detection loss.

## Figures and Tables

**Table 1:** Chonnam National University Hospital (CNUH) dataset.

Knee Region	Benign Tumor	Malignant Tumor	Normal
Distal femur	598	89	-
Proximal tibia	463	45	-
Total	1061	134	381

**Table 2:** Ablation study to specifically test the effects of the three branches with global, patch-based, and combination approaches.

No.	Model	Classification	Segmentation	Multi-Level Distance	Patch	Global
1	Seg-Unet		🗸			
2	Seg-Unet + ClasSeg	🗸	🗸			
3	Seg-Unet + ClasSegDis Patch	🗸	🗸	🗸	🗸	
4	Seg-Unet + ClasSegDis Global	🗸	🗸	🗸		🗸
5	Seg-Unet + ClasSegDis Patch + Global	🗸	🗸	🗸	🗸	🗸

**Table 3:** Segmentation results of CNUH dataset on the validation set.

No	Model	*MeanIoU*
1	Seg-Unet	69.50%
2	Seg-Unet + ClasSeg	77.28%
3	Seg-Unet + ClasSegDis Patch	66.53%
4	Seg-Unet + ClasSegDis Global	78.89%
5	Seg-Unet + ClasSegDis Patch + Global	**84.84%**

**Table 4:** Classification results of the CNUH dataset on the validation set.

No	Model	*Accuracy*	*Mean* ± *stdAccuracy*	*F*_1_
2	Seg-Unet + ClasSeg	95.27%	82.27% ± 29.60%	94.57%
3	Seg-Unet + ClasSegDis Patch	77.29%	80.37% ± 13.72%	78.58%
4	Seg-Unet + ClasSegDis Global	94.32%	93.97% ± 5.61%	94.42%
5	Seg-Unet + ClasSegDis Patch + Global	**99.05%**	**96.30%** ± 6.41%	**99.03%**

**Table 5:** Performance comparison with related studies on the CNUH validation set.

No.	Model	*Accuracy*	*MeanIoU*
1	MobileNet V2 [12]	93.60%	
2	VGG16 [13]	90.50%	
3	RSS-BW with VGG16-B [14]	86.93%	
4	U-Net [8]		38.30%
5	Seg-Net [9]		57.10%
6	Seg-Unet [15]		69.50%
7	Seg-Unet with Clas. and Seg. [7]	95.30%	77.28%
8	Seg-Unet with Clas., Seg., and distance features [16]	97.16%	78.83%
9	Our proposed method (Patch)	77.29%	66.53%
	Our proposed method (Global)	94.32%	78.89%
	Our proposed method (Gloal + Patch)	**99.05%**	**84.84%**

**Figure A1:**
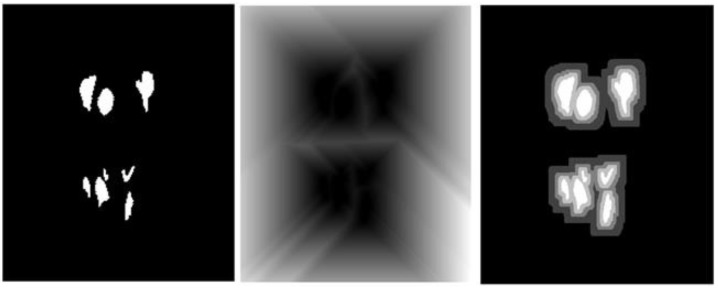
(**Left**) Tumor mask, (**Middle**) Distance transform, and (**Right**) Distance Mask.

**Figure A2:**
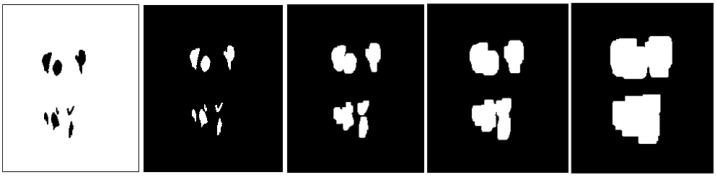
Multi-level distance mask.

**Figure A3:**
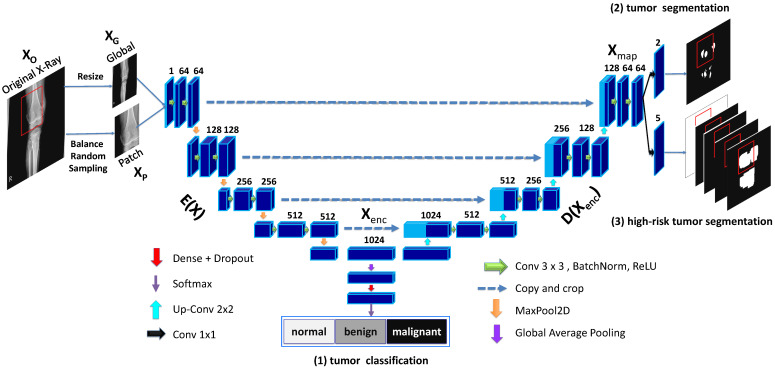
Multi-level Seg-Unet model with global and patch-based approaches.

## Data Availability

N/A.

## Appendix A. Related Works

In medical image research, any determinations of lesions and abnormalities must be made with a high level of accuracy, which also demands precise segmenting of these regions. Three typical approaches to medical image segmentation are manual segmentation, semi-automatic segmentation, and automatic segmentation. Manual segmentation demands experienced experts as well as a lot of time and cost. In the semi-automatic approach, users must provide some inputs to support the segmentation process [19]. Finally, the automatic segmentation is a fully automatic method without user input, but it is difficult to obtain accurate results by relying solely on the machine. However, this is a unique feasible method for applications involving a substantial number of images.

### Appendix A.1. Tumor Detection

There are several typical works related to medical image processing such as Li et al.’s [20] study of liver cancer, which is one of the deadliest types of cancer; Esteva et al.’s [21] exploration of skin cancer aiming to classify skin lesions using images alone; and Milletari et al. [22], who proposed a 3D medical image segmentation model and trained it end-to-end on MRI volumes depicting the prostate. In traditional medical image processing techniques, researchers leveraged image features to extract texture descriptions as local information. In particular, the authors of [23] suggested a hybridized approach of edge and region-based techniques, while Abdel-Maksoud et al. [24] used unsupervised learning, including K-Means and Fuzzy C-Means algorithm, for brain tumor segmentation.

Recently, many deep learning methods have been introduced that can accurately handle medical image classification and segmentation, and many new network architectures have emerged. In 2015, the U-Net architecture [8] was introduced to segment biomedical images. This network can be trained end-to-end and achieve better results than traditional methods like the sliding-window convolution network. In 2018, Li et al. [20] proposed H-DenseUNet, a hybrid densely connected UNet-like with 2D and 3D DenseUNet.

To deal with the high-resolution of medical images, Ronneberger et al. [8] developed a model based on a U-net model as well as an overlap strategy to handle arbitrary large images through seamless segmentation. Li et al. [20] used connections between layers to maximize and ensure the information during the training process. Then, they used a sliding window strategy on the image patches of the original image to predict the tumor regions.

### Appendix A.2. Knee Bone Tumor Detection

A lot of prior studies involving radiographic image segmentation of the human knee have only focused on knee osteoarthritis assessment [25,26] or knee bone detection [27,28]. However, there is very little research applying radiographic images to segment knee bone tumors: George et al. [29] used various texture features of radiography to recognize bone patterns in the tumor region. In [17], Do et al. applied a Bayesian classifier to identity bone tumor diagnoses based on a combination of radio-graphic observations and demographic characteristics.

Moreover, Reicher et al. [5] used a deep learning method to classify the bone tumor matrix; the highly accurate result shows the importance of the bone tumor matrix in bone tumor diagnosis. In 2019, Ho et al. [14] used the bidirectional W-network to segment three knee bone regions to input them into the semi-supervised bidirectional W network to classify tumor types.

## Appendix B. Implementation Details

### Appendix B.1. Multi-Level Distance Features

The distance transform is defined as the associating function between a set of points *P* to each grid location *q* by the nearest point in *P* to *q* as follows:

(A1) $$D_{P}\left(p\right)=\min_{q\in P}\left\{d\left(p,q\right)+f\left(q\right)\right\}$$

where $d\left(p,q\right)$ is the distance between p and q, and f is a function on the grid containing *q*.

We choose the pixels in the tumor regions as *P* with the Euclidean distance measure and *f* as the membership indicator function of *P* with 0 when q∈P and inf otherwise. Then, we apply a threshold for the result from the distance transform after normalizing the values to [0, 1] in the following manners:

(A2) $$T\left(d\right)=\left\{\begin{array}{cc} 1 & d=0\\ 0.75 & d<0.25\\ 0.5 & d<0.5\\ 0.25 & d<0.75\\ 0 & otherwise \end{array}\right.$$

To calculate the tumor distance mask in Figure A1, we apply the distance transform on the tumor mask, then the threshold by four distances as shown in Equation (A2).

The multi-level distance masks are five-level masks with non-tumor regions, and the tumor distance mask in the specific threshold as shown in Figure A2:

The role of multi-level distance masks is to help the network capture the semantic information around the tumor regions.

### Appendix B.2. Loss Function

With the classification branch, we use categorical cross-entropy loss. The segmentation and distance branches are applied using the dice loss equation as follows:

(A3) $${£}_{dice}=1-\frac{\sum_{i=1}^{N}2\left|y_{i}{\hat{y}}_{i}\right|+\epsilon }{\sum_{i=1}^{N}\left(left|y_{i}\right|+\left|{\hat{y}}_{i}\right|)+\epsilon }$$

where *N* is the amount of segmentation labels, ${\hat{y}}_{i}$ is the predicted mask compared to the ground-truth $y_{i}$, and ϵ is the smooth term.

Finally, the multi-task loss in our network is expressed as follows:

(A4) $$£=\alpha_{1}{£}_{clas}+\alpha_{2}{£}_{seg}+\alpha_{3}{£}_{dis}$$

where ${£}_{clas}$, ${£}_{dis}$, and ${£}_{seg}$ are respectively the classification, segmentation, and distance losses with $\alpha_{1}=\alpha_{2}=\alpha_{3}=1$ as balancing parameters.

### Appendix B.3. Fusion of Global and Patch-Based Models

Global-based model. The global-based model has the Seg-Unet architecture shown in Figure A3. Its input is the scaled-down image from the high-resolution original image, which is used to learn the geometric features from the whole image. Its goal is to provide a classification result determining where the image should be labelled as normal, benign, or malignant, as well as a segmentation result for identifying the tumor regions in the image. This integration between two tasks leads to a regularizing effect on the learning process by the sharing of features, which makes them improve together. This means that the classification task can make more precise predictions based on the segmentation information. Otherwise, when knowing the result of the classification task, the segmentation task can segment quickly using the global-context information with normal, benign, or malignant classification. Using the multi-level distance feature map in segmentation branch, the global model can detect small tumor regions in the X-ray image. However, it can also be inaccurate in difficult cases based on the scaled-down effect from the original image.

Patch-based model. The patch-based model uses the pre-training weight of the global model to transfer the knowledge of the global context. From there, it learns more details of the texture features from small image chunks of the original image. Every high-resolution image will be randomly cropped into small image chunks to feed into the patch-based model. We generate image chunks with a balance between the numbers of tumor and non-tumor region occurrences. Image chunks containing tumor regions that are too small under the specific number of pixels (decided in the experiment) will be labelled as normal image chunks.

In the prediction process, the segmentation result of the overall X-ray image is calculated by choosing the maximum tumor probabilities of all image chunks in the whole image with a sliding window. A sliding window is used to generate all image chunks from left to right and from top to bottom, where it can create overlapping regions. We normalize the whole probability map into the range [0, 1], obtain a specific threshold for detecting tumor regions, and apply post-prepossessing by eliminating very small regions below the specific number of pixels (about 500 pixels).

For the classification region, the role of the patch-based model is to focus in detail on occurrences of regions with high-risk tumor probability. Therefore, we use a max operator to integrate all tumor probabilities in the classification prediction of all image chunks containing tumor regions. This means that, if one of the image chunks containing tumor regions in the X-ray image has a high probability of tumor classification, the whole image will be labelled as high-risk in tumor occurrence. This helps reduce the normal prediction from the normal image chunks. If the image has no tumors, the classification probability will be calculated by the max operator from all possible image chunks in an X-ray image.

Fusion method. The fusion approach takes advantage of both the global and patch-based models to boost the overall performance results. The segmentation task proceeds as follows: Let $G_{ij}$ and $A_{ij}$ be the segmentation probabilities in the global and patch-based models, respectively, at position $\left(i,j\right)$, where $G_{ij}$ and $A_{ij}$ are one-hot vectors with normal and tumor probabilities, and we will calculate the segmentation probabilities $C_{ij}$ in the fusion approach as follows:

(A5) $$C_{ij}=\beta G_{ij}+\left(1-\beta \right)A_{ij}+\left(1-\beta \right)A_{ij}K_{ij}$$

where β is the balance factor used to adjust the priority between the global and patch-based models. In this study, we chose $\beta =\frac{2}{3}$ to take priority in choosing the segmentation result from the global-based model. $K_{i,j}$ is the binary mask of the tumor regions from the patch-based model adjacent to the tumor region in the global-based model, and they do not belong to the intersection between the tumor regions according to the global and patch-based models.

We determined four cases to calculate the weighted value at pixel $\left(i,j\right)$. We gave priority to the intersection of tumors in the global and patch-based models (case 1) with $\beta G_{ij}+\left(1-\beta \right)A_{ij}$. Next, the probability values of the tumor regions determined by the global-based model (case 2) and patch-based model (case 3) are $\beta G_{ij}$, and $\left(1-\beta \right)A_{ij}$, respectively. To identify the tumor regions of the patch-based model expanding the tumor regions of the global-based model (case 4), we used $K_{ij}$ and we calculated the probability with $2*\left(1-\beta \right)A_{ij}$. Therefore, the priority order of the four cases is cases 1, 2, 4, and 3. Finally, we normalize the fusion of the probability map into the range $\left[0,1\right]$.

For the classification outputs, the global-based model can robustly distinguish tumor/normal regions by capturing the whole image to learn the geometric and global-context characteristics. It also achieves good results in tumor classification between benign and malignant due to the multi-level distance feature map. However, it faces issues in difficult cases, such as when the tumor size is very small against the high-resolution of the image input, which leads to information loss when reducing the image size.

By contrast, the patch-based model generated the classification probabilities based on the maximum operator of the image chunks containing tumor regions. Therefore, it addresses the high-resolution challenge in difficult cases; however, it often produces more noise in segmentation and fails to distinguish between normal and tumor images using normal X-ray images.

Based on the above analysis, we integrate the results of the global and patch-based models in the classification as follows:

(A6) $$\begin{array}{c} \begin{array}{cc} P_{i}^{c\neq c_{n}} & =\beta *G_{i}^{c\neq c_{n}}+\left(1-\beta \right)A_{i}^{c\neq c_{n}}\\ P_{i}^{c_{n}} & =\left\{\begin{array}{cc} \beta *G_{i}^{c_{n}}+\left(1-\beta \right)A_{i}^{c_{n}} & if\left|\sum_{c\neq c_{n}}G_{i}^{c}-G_{i}^{c_{n}}\right|\leq T\\ 0 & otherwise \end{array}\right.\\ where\left|P_{i}\right|=1 \end{array} \end{array}$$

where $G_{i}^{c}$ and $A_{i}^{c}$ are the classification probability vector of image *i*; *c* is the classification label with three types among benign, malignant, and normal, where $c_{n}$ is the normal label; and β is the control factor adjusting the priority level between both models. We will take priority over the global-based model by choosing $\beta =\frac{2}{3}$ in the classification probability calculation. However, the probability of the normal label will be set to 0 if the total probability of malignant and benign in global-based reach the normal probability by the threshold *T* value. All fusion probability vectors will be normalized to 1.

## Appendix C. Environment Setup and Evaluation Metrics

**Environment Setup**. To implement our method, we used the Keras and TensorFlow framework on the environment Python 3.7. We conducted our experiments on a desktop machine with the following hardware: Intel Core i7 8700, 64 GB RAM, and two NVIDIA GTX1080 Ti graphic cards with 11 GB RAM.

**Evaluation Metrics**. Our study used Accuracy, $F_{1}$ score, $Mean_{acc}$, and $Std_{acc}$ for classification evaluation, where $Mean_{acc}$ and $Std_{acc}$ are, respectively, the average and standard deviation of the percent values on the main diagonal of the confusion matrix. These metrics are calculated as follows:

(A7) $$\begin{array}{c} \begin{array}{cc} Accuracy & =\frac{TP+TN}{TP+FP+TN+FN}\\ F_{1} & =2\frac{Precision.Recall}{Precision+Recall}\\ Mean_{Acc.} & =\frac{\sum_{i=1}^{n}G_{i,i}}{n}\\ Std_{Acc.} & =\sqrt{\frac{\sum_{i=1}^{n}{\left(G_{i,i}-Mean_{Acc.}\right)}^{2}}{n}} \end{array} \end{array}$$

where TP, TN, FP, and FN are the true positive, true negative, false positive, and false negative values, respectively. $G_{i,i}$ is the percent values on the main diagonal of the confusion matrix. Precision and Recall are respectively calculated as follows:

(A8) $$\begin{array}{c} \begin{array}{c} Precision=\frac{TP}{TP+FP}\\ Recall=\frac{TP}{TP+FN} \end{array} \end{array}$$

where Precision and Recall evaluate the numbers of correct predictions for all positive samples and for true samples, respectively.

Accuracy measures correct prediction, so it only demonstrates the performance of the model when there is an almost symmetric data distribution on training and validation. To evaluate under an uneven imbalance distribution, we used $F_{1}$ score and $Mean_{acc}\pm Std_{acc}$.

For tumor segmentation and high-risk tumor segmentation, we used the MeanIoU metric [30] for the quantitative measurements. First, the metric calculates the average of the intersection over union between the ground-truth and all segmentation results on every X-ray image. Next, it averages all MeanIoU values of the whole X-ray images in evaluation data. The following equation is used for this:

(A9) $$\begin{array}{c} \begin{array}{cc} MeanIoU\left(I\right) & =100-\frac{1}{\left|C\right|}\sum_{c\in C}\frac{2\sum_{p\in I}p_{c}{\hat{p}}_{c}+\epsilon }{p_{c}+{\hat{p}}_{c}+\epsilon }100\\ MeanIoU\left(D\right) & =100-\frac{1}{K}\sum_{k=1}^{K}MeanIoU\left(I_{k}\right)100 \end{array} \end{array}$$

where *I* and $I_{k}$ are the images of validation data *D* with the number of images *K*; *C* is the number of labels; $p_{c}$ and ${\hat{p}}_{c}$ are the ground-truth and prediction pixels, respectively, in class *c*; and ϵ is the smoothness term to avoid zero division.
